# New Antenatal Model in Africa and India (NAMAI) study: implementation research to improve antenatal care using WHO recommendations

**DOI:** 10.1186/s12961-023-01014-5

**Published:** 2023-08-10

**Authors:** NAMAI Research Group, Seni Kouanda, Adja M. Ouedraogo, Armel E. Sogo, Ouahabou Bagaya, Tilado E. V. Sorgho, Yelmali C. Hien, Dina V. Gbenou, S. Ramatou Sawadogo Windsouri, Wilfried Zoungrana, Moussa Dadjoari, Valerie M. Zombré Sanou, Gulnoza Usmanova, Yashpal Jain, Ram Chahar, Somesh Kumar, S. V. Vinod Kumar, Ashish Srivastava, Tapas Sadasivan Nair, Abu H. Sarkar, Nitin Bajpai, Vaibhav Patwardhan, Chandra Shekhar Joshi, Manish Chotiya, Dinesh Baswal, Sabine Musange, Felix Sayinzoga, Vincent Mutabazi, Nathalie K. Murindahabi, David Nzeyimana, Bernard Ngabo Rwabufigiri, Theopista J. Kabuteni, Maria Mugabo, Michael Mbizvo, Caren Chizuni, Nachela Chelwa, Rosemary Muliokela, Chifundo Phiri, Kondwani Kasonda, Natasha Okpara, Muyereka Nyirenda, Sarai B. Malumo, Angel Mwiche, Virginia Simushi, Nathan Nsubuga Bakyaita, María Barreix, Özge Tunçalp, Soe Soe Thwin, Maurice Bucagu, Tigest Tamrat, Ndema Habib, Amnesty E. Lefevre, Fabiana Lorencatto

**Affiliations:** UNDP/UNFPA/UNICEF/WHO/World Bank Special Programme of Research, Development and Research Training in Human Reproduction (HRP), Department of Sexual and Reproductive Health and Research, World Health Organization, Avenue Appia 22, 1211 Geneva, Switzerland

**Keywords:** Antenatal care, Implementation research, Maternal health, Newborn health, Digital interventions, Respectful maternity care, Digital health, Health systems

## Abstract

**Background:**

In 2020, an estimated 287 000 women died globally from pregnancy‐related causes and 2 million babies were stillborn. Many of these outcomes can be prevented by quality healthcare during pregnancy and childbirth. Within the continuum of maternal health, antenatal care (ANC) is a key moment in terms of contact with the health system, yet it remains an underutilized platform. This paper describes the protocol for a study conducted in collaboration with Ministries of Health and country research partners that aims to employ implementation science to systematically introduce and test the applicability of the adapted WHO ANC package in selected sites across four countries.

**Methods:**

Study design is a mixed methods stepped-wedge cluster randomized implementation trial with a nested cohort component (in India and Burkina Faso). The intervention is composed of two layers: (i) the country- (or state)-specific ANC package, including evidence-based interventions to improve maternal and newborn health outcomes, and (ii) the co-interventions (or implementation strategies) to help delivery and uptake of the adapted ANC package. Using COM-B model, co-interventions support behaviour change among health workers and pregnant women by (1) training health workers on the adapted ANC package and ultrasound (except in India), (2) providing supplies, (3) conducting mentoring and supervision and (4) implementing community mobilization strategies. In Rwanda and Zambia, a fifth strategy includes a digital health intervention. Qualitative data will be gathered from health workers, women and their families, to gauge acceptability of the adapted ANC package and its components, as well as experience of care. The implementation of the adapted ANC package of interventions, and their related costs, will be documented to understand to what extent the co-interventions were performed as intended, allowing for iteration.

**Discussion:**

Results from this study aim to build the global evidence base on how to implement quality ANC across different settings and inform pathways to scale, which will ultimately lead to stronger health systems with better maternal and perinatal outcomes. On the basis of the study results, governments will be able to adopt and plan for national scale-up, aiming to improve ANC nationally. This evidence will inform global guidance.

Trial registration number: ISRCTN, ISRCTN16610902. Registered 27 May 2022. https://www.isrctn.com/ISRCTN16610902

## Background

In 2020, an estimated 287 000 women died globally from pregnancy‐related causes and 2 million babies were stillborn, half occurring during the third trimester [1, 2]. Many of these adverse outcomes can be prevented by quality healthcare during pregnancy and childbirth. Within the continuum of maternal health, antenatal care (ANC) is a key moment in terms of contact with the health system, yet it remains an underutilized platform [3, 4]. Implementing timely and appropriate evidence‐based practices during ANC can improve maternal and fetal health [5, 6]. Recent evidence shows that while most (more than 80%) women receive at least 1 ANC visit, only 49.3% receive 4–7 visits, and 11.3% received 8 ANC visits in low- and middle-income countries (LMICs) [7]. This was especially due to late initiation, as most women (50.1%) had their first visit after their first trimester [8].

To improve maternal health and outcomes, the WHO released its comprehensive recommendations on ANC for positive pregnancy experience in 2016 [9]. The guideline is intended to prioritize person-centred care and wellbeing, not only the prevention of death and morbidity [10]. It includes recommendations related to antenatal nutrition, maternal and foetal assessments, preventative measures, interventions for common physiological symptoms (e.g. nausea, heartburn, constipation), as well as health systems interventions to improve ANC utilization and quality of care for a consolidated package of routine ANC. Despite the value of guidelines for setting standards in healthcare provision, applying these recommendations within local contexts and at point of care is a well-documented challenge, including for maternal health [11–16]. Additionally, variability and complexity of local conditions require adaptations of the global guidelines, thus driving WHO to develop new efforts to assist countries in adapting and implementing the ANC recommendations. Finally, the use of implementation science, informed by theories and frameworks, to help promote evidence-based interventions in LMICs, has been limited [17, 18].

Since February 2018, the governments of Burkina Faso, Rwanda and Zambia, as well as Assam and Tamil Nadu states in India, have prioritized the adaptation and adoption of the WHO ANC recommendations. Initial efforts lead to the development of the WHO ANC recommendations adaptation toolkit [19]. Governments and stakeholders employed the toolkit to adapt the ANC package and update national- and state-level ANC policies. As a next step, this protocol outlines implementation research, applying the Capability Opportunity Motivation-Behaviour (COM-B) model for behaviour change to design co-interventions, and the theoretical framework of acceptability, to better understand what it will take to provide quality, integrated and person-centred ANC packages [20, 21]. In Rwanda and Zambia, this will include the deployment of a country-adapted digital intervention (WHO digital ANC module) to support the implementation of their ANC packages [22], in line with national digital health strategies [23] and the WHO’s SMART guideline approach [24].

In collaboration with Ministries of Health (MoHs) and country research partners, the study uses implementation science to systematically introduce and test the applicability of the adapted ANC package in selected sites across four countries. It aims to generate key evidence on the implementation of the adapted ANC packages and inform future national adaptation and scale up. Study objectives are outlined in Table 1; in Rwanda and Zambia, there will be a formative phase.

**Table 1:** Study objectives

Burkina Faso and India
*Primary objectives*
1. Does the adherence to the country/state package of ANC interventions by health workers improve with the country/state-specific ANC package? a. Does the implementation of country/state-specific ANC package (including targeted community mobilization) increase early initiation of ANC among pregnant women? 2. Is the country/state-specific ANC package acceptable to and does it create value for women and health workers?
*Secondary objectives*
1. What are the human resources training and supervision needs of implementing the country/state-specific ANC package? 2. What is the cost of implementing the country/state-specific ANC packages compared with standard of care? 3. What are the effects of the ANC package on retaining women in ANC services? (Burkina Faso only)

Rwanda and Zambia
*Formative phase*
1. Adapt the WHO digital ANC module to the country-specific ANC package and local healthcare delivery context, customizing the ANC Digital Adaptation Kit (DAK) [36]
2. Identify bugs and errors in the clinical algorithms and in the country-adapted ANC digital module
3. Gather general feedback regarding the usability of the country-adapted ANC digital module and components for improvement
4. Develop and test training materials for the country-adapted ANC digital module
5. Develop and test supervision and technical support mechanisms for the country-adapted digital ANC module
*Demonstration phase—primary objectives*
1. Does the adherence to the country/state package of ANC interventions improve among health workers using the adapted WHO digital ANC module compared with standard care? a. Does the implementation of the country-specific ANC package (including targeted community mobilization) increase early initiation of ANC among pregnant women? 2. Is the use of the country-adapted WHO digital ANC module acceptable to, and does it create value for, women and health workers?
*Demonstration phase—secondary objectives*
1. What are the human resources training and supervision needs to ensure fidelity of deployment of the adapted WHO digital ANC module? 2. What are the financial resources needed to implement the adapted WHO digital ANC module for the study, and what resources would be needed to achieve scale?

## Methods: description

The proposed study is composed of two layers of interventions:

1. The first refers to the ANC package adapted by each country or state (in India the study will be conducted in Assam and Tamil Nadu states). The adapted ANC packages are based on the WHO 2016 ANC recommendations. Each country or state adapted the WHO recommendations through a standardized process, which included conducting a situational analysis and stakeholder consultations to meet local needs and context. [19]
2. The second refers to the co-interventions (or implementation strategies) to support the implementation of the adapted ANC package. The study’s co-interventions, the ‘how to’ components of changing health service provision [25], seek to modify the behaviour of both pregnant women and health workers, to ultimately improve ANC service provision. The co-interventions address findings from Downe et al.’s Cochrane review on qualitative evidence on the provision and uptake of routine ANC services [26]. In Rwanda and Zambia, this will include a digital health component.

### Logic model

A logic model was developed to specify the components of the co-interventions and how they are proposed or hypothesized to bring about changes in ANC service delivery (including improving implementation of the adapted ANC package) and uptake. We drew on the COM-B model of behaviour change and the related, Behavior Change Wheel, employed to conceptualize and describe the behaviour change targeted by the co-interventions [20]. COM-B proposes three necessary conditions for a desired behaviour to occur: (1) the person’s psychological and physical ability to perform it (capability – that is, having the necessary knowledge and skills), (2) their reflective and automatic mechanisms which ‘energize’ or prevent it (motivation – that is, strong intention to perform the behaviour, perceived importance, priority, advantages and disadvantages) and (3) the external social and environmental opportunity which enables or inhibits it (opportunity – that is, available resources, team culture and working norms) [20].

**Fig. 1:**
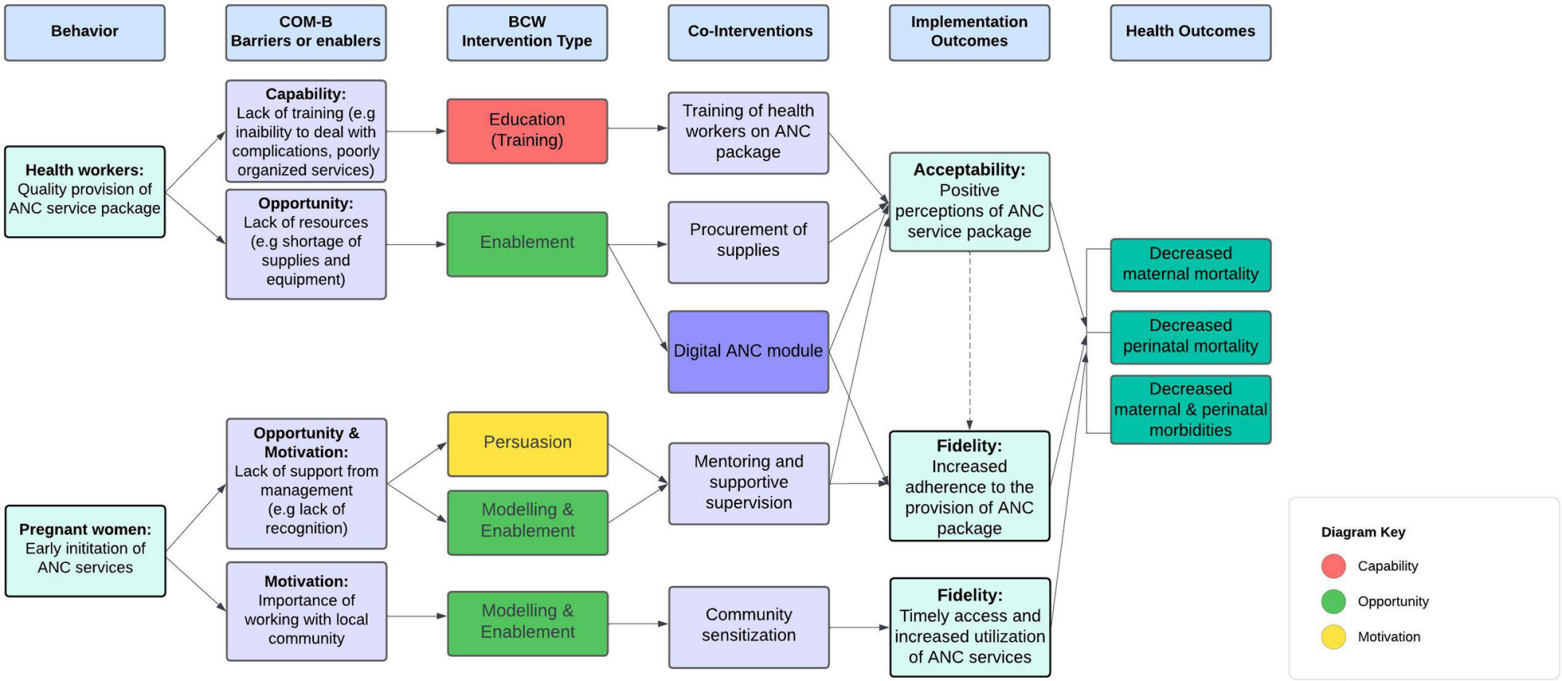
Study logic model. The colours in the third column are associated with either: capability (red), motivation (yellow) or opportunity (green). The purple box applies only to Rwanda and Zambia. COM-B: Capability Opportunity Motivation-Behavior ; BCW: Behavior change wheel

Each co-intervention addresses one of these conditions. Training health workers will build health workers’ capability through increasing knowledge, skills, and understanding of the adapted ANC package. Providing the necessary supplies and equipment will reduce barriers and increase health workers’ opportunity (physical) to implement the ANC package. In Rwanda and Zambia, employing the country-adapted digital module will support health workers’ capability, enabling them to adhere to the provision of the ANC package. Mentoring and supervision will provide hands on learning opportunities (social) and examples to imitate, as well as increase motivation through persuasion. Community mobilization will impact social influences on women, their families and key community stakeholders through persuasive communication. Better trained and supported health workers and available supplies aim to increase the positive experiences and perceptions that women have of ANC services, increasing their demand. Additionally, community-level messaging will focus on the importance of ANC seek to influence and encourage women and their families to access ANC early and more frequently. Figure 1 outlines and categorizes the four study co-interventions, linking them to the relevant COM-B domain and study outcomes.

### Study design – India & Burkina Faso

**Fig. 2:**
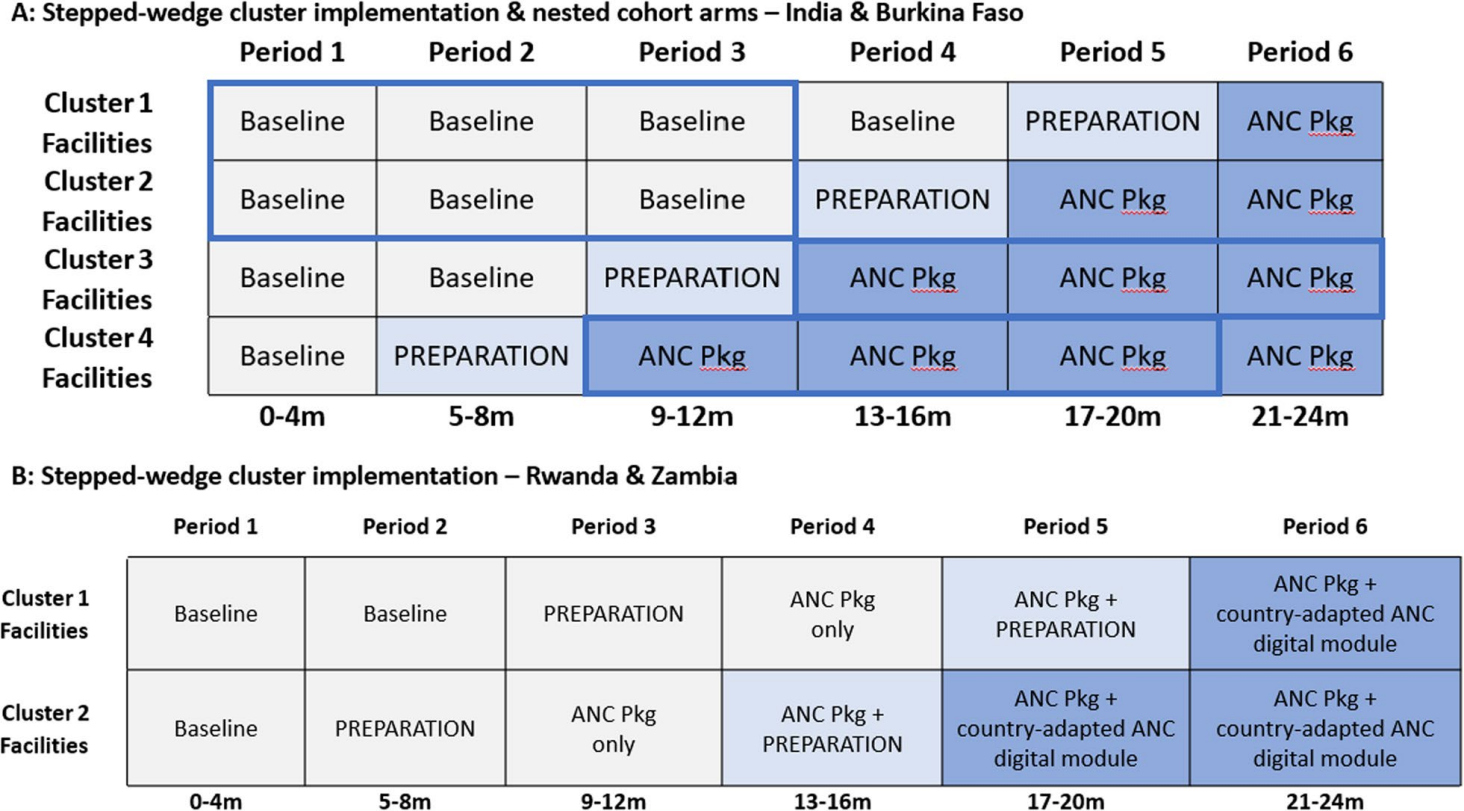
**A**, **B** Stepped-wedge cluster implementation designs. *Pkg* package, *ANC* antenatal care. The blue outlines refer to the nested cohort recruitment and follow up periods

The study design is a mixed methods stepped-wedge cluster randomized design implementation trial with nested cohort study (in India and Burkina Faso) (Fig. 2A). The design involves a sequential crossover of clusters from the control to the intervention arm, so that every cluster, composed of selected primary health facilities, begins in the control condition and eventually receives the intervention, with random assignment to the order of crossover.

Health facilities will be randomly allocated to one of four clusters. The study period of 24 months will be divided into six periods of four months each. All selected facilities will cycle through the following steps:

- Baseline phase: Facilities will all begin with at least one four-month period of baseline data collection
- Preparation phase: This will be a transition phase where all the co-interventions will be initiated.
- Full implementation phase: Depending on the cluster [1–4], health facilities will implement the ANC package for a range of either one to four periods (4–16 months). Please see Fig. 2A for more information.

Implementation of the adapted ANC package will be done cluster wise from period 2 onwards, such that by period 6 all clusters would have received the intervention. Data will be collected cross-sectionally at the end of each month in each cluster.

In addition to collecting cross-sectional data in each cluster every period, two cohorts of pregnant women will be enrolled and followed through the course of their pregnancy. One cohort will not be exposed to the intervention (comparison arm). For the comparison group, pregnant women in cluster 1 and cluster 2 will be enrolled during the first three months of period 1 and followed through to the end of their pregnancy (period 3). For the intervention group, pregnant women in clusters 3 and 4 will be enrolled. To ensure comparability, for cluster 3, women will be enrolled during the first three months of period 4 and followed through to the end of their pregnancy (period 6), while for cluster 4, women will be identified and consented during the first three months of period 3 and followed through to the end of their pregnancy (period 5). Purposive sampling will be used to identify women coming for their first ANC during the first trimester. Research teams will monitor selection to ensure a balance in maternal age, parity, education level and religion.

### Study design – Rwanda & Zambia

The study will be implemented in two sequential phases. The first is a formative phase (approximately 6 months) to establish operational requirements and assess the country-adapted ANC digital module’s functionality. The WHO digital ANC module is a tool based on WHO-guideline-derived algorithms that provides clinical decision support and person-centric management, including tracking and patient records, to assist health workers in implementing the ANC package [22]. The MoHs and country teams conducted a landscape analysis to prioritize integration of the country-adapted module to existing digital systems (e.g. DHIS 2, etc.) and customized the ANC Digital Adaptation Toolkit (DAK) [23]. During the formative phase, the WHO reference software will be adapted to create the Rwanda/Zambia ANC digital module. Qualitative methods will be employed to gauge health workers’ ability to use the module. A training package for ANC health workers [27] and related support and supervision materials will be tailored for the study. The formative component will not be carried out in health facilities that are part of the demonstration phase, but in those that share similar characteristics.

The second, demonstration phase (24 months), employs a mixed methods stepped-wedge cluster randomized design implementation trial to test the introduction of the adapted ANC package and the digital tool in selected health facilities in both clusters (Fig. 2B). Facilities will be pair-matched on the basis of factors affecting ANC enrolment, quality and facility capacity. Within each pair, a facility will be randomly chosen to start implementing first the adapted ANC package, and then the adapted ANC digital module. All selected facilities will cycle through the following steps:

- Baseline phase: Facilities will all begin with at least one four-month period of baseline data collection. Cluster 1 facilities will collect an additional four months of baseline data.
- Preparation phase for the deployment of the national package of ANC interventions: This will be a transition phase where all co-interventions, except the digital module, will be initiated. 
- Adapted ANC package implementation phase: Health facilities will then have four months to implement the adapted ANC package.
- Adapted ANC package implementation and preparation for deployment of country-adapted ANC digital module: This will be a transition phase where the digital co-intervention will be initiated; the other four co-interventions will continue to be implemented.
- Adapted ANC package and adapted ANC digital module implementation: Facilities will deploy both the adapted ANC package and the digital tool to assist health workers in implementing the former.

### Target population

Sub-national locations (i.e. region, state, districts, province, blocks) were selected in collaboration with the MoHs. For example, in some instances, the MoH considered locations with high or low performance^1^ on maternal and child health indicators to ensure that learnings will be applicable to settings with varying level of development. Generally, health facilities need to have a minimum volume of 30 women attending ANC once per month, human resource capacity of minimum two health workers providing ANC, and physical infrastructure (e.g. separate room dedicated to ANC service delivery).

Additionally, the following exclusion criteria were applied:

- Health facilities where the management does not agree to participate in the study,
- Health workers who do not consent (qualitative components),
- Women who do not provide consent (all study components),
- Women who do not reside in the catchment area of selected healthcare facilities (Burkina Faso and India),
- Women who also sought ANC services from non-public health platforms, such as the private sector (Rwanda),
- Girls under 14 years old (Burkina Faso & India),
- For the cohort study:
  - women at more than 20 weeks of gestation,
  - women with pre-identified complications.

### Co-interventions

Country study teams are charged with deploying the co-interventions. Teams are made up of the MoH, an implementation research partner^2^ and the WHO country offices. WHO regional and headquarters offices will be involved in the overall coordination across the four countries and support in the monitoring and evaluating the study and summarizing global results. In Rwanda and Zambia, the MoH selected a local technical partner to support the digital tool adaptation; additionally, the partner that developed the WHO digital ANC module will provide support and knowledge transfer throughout the project.^3^

#### Co-intervention 1: training of selected primary healthcare staff on adapted ANC package

WHO AFRO developed training resources (including facilitator guides and presentations, etc.) for the WHO ANC recommendations. Each country team will tailor these resources for health workers who are involved in the provision of ANC services at selected health facilities. In India, Jhpiego resources were also incorporated into the training materials. Where appropriate, a Training of Trainers (ToT) model will be applied. Thereafter, ANC health workers [and in some cases, catchment area staff, such as community health workers (CHWs)] will be trained on the adapted ANC package during 3–5 day training and orientation sessions. Where applicable, existing ANC registers at selected study facilities (and catchment areas) and client cards issued to all pregnant women used for recording ANC services will be modified to capture service data according to the adapted ANC package. The training and supervision sessions (see co-intervention 2) will include capacity building of health workers on these modified recording tools.

In Burkina Faso, Rwanda and Zambia, nurses and/or midwives providing ANC service in study facilities will be trained to conduct ultrasounds (before 24 weeks of gestation). Country teams will develop a package to train primary health workers to use and interpret ultrasound machines and results. A team made up of obstetricians/gynaecologists and/or radiologist technicians will conduct the trainings, which will include an introduction to theory and a practice component. Study facilities will select at least one ANC health worker to attend. Results from the training evaluation will assist in identifying components that will require continuous support through supervision.

#### Co-intervention 2: supervision needs to support staff and ensure fidelity of deployment of adapted ANC package

Country teams (possibly including relevant district and/or national focal persons) will conduct structured supervision visits to all study facilities. Where applicable, mentoring of facility staff will also be carried out during this process. Standardized tools will be employed during supervision visits. These visits will include support and mentoring of ultrasound trainees (except in Indian states) and on the use of the ANC digital tool (Rwanda and Zambia only). For the latter, the local technology partner and country team will ensure that the health workers using the digital tool are adequately supported.

#### Co-intervention 3: availability of supplies for country/state-level ANC package

Country teams will conduct facility assessments at each site, once every four months, to monitor the availability of supplies (drugs, supplements, testing kits, equipment, etc.) which are required for implementing the adapted ANC package. They will use a standard facility assessment checklist for carrying out the assessments. If needed, the country team will reach out to the respective local-level authorities to ensure that supplies are replenished in time and there are no stockouts. Additionally, where appropriate, the implementation research partner will provide supplies (from a buffer stock) to ensure the ANC package can be implemented.

#### Co-intervention 4: community mobilization on country/state-level ANC package

Existing community mobilization activities will be harnessed to generate awareness on the adapted ANC package among pregnant women and their communities. Depending on the setting, this will include CHWs and/or community leaders and/or maternal health groups or committees serving in the study sites’ catchment areas. A scoping review of community mobilization activities for ANC uptake and early ANC was conducted to inform and support this process [28]. Activities to distribute messaging regarding the adapted ANC package may include conducting regular community-level sensitization meetings, home visits to pregnant women, facilitation of educational talks during maternal support groups, etc. Country teams will use information, education and communication materials such as flip charts, posters, etc. (as applicable) for conducting these activities. Where CHWs are involved, country teams may include them in their trainings and supervision visits to the study sites.

#### Co-intervention 5: (Rwanda & Zambia only) training of select health centre staff on the country-adapted ANC digital module

To train health workers in the use of the country-adapted ANC digital module, a generic training resource package (including facilitator guides and presentations, etc.) has been developed and adapted for use in each country. ANC health workers will use smartphones for service provision. Country teams will apply a ToT model where appropriate.

## Methods: evaluation

### Quantitative data (adherence to ANC package and early initiation of ANC)

The primary outcomes or indicators of interest, which will be measured to assess the impact of the adapted ANC packages, vary across each country (see Table 2). These measures are based on the WHO ANC monitoring framework [29].

**Table 2:** Primary outcomes by country

	Burkina Faso	India	Rwanda	Zambia
Syphilis testing at first ANC contact	X	X	X	X
First ANC contact within 12 weeks	X	X	X	X
Iron and folic acid (IFA) supplementation received for 90+ days	X			
Haemoglobin (Hb) screened at first ANC contact		X	X	X
Total number of ANC contacts	X	X		
Blood pressure measurement at first ANC contact				X

X denotes which primary outcomes are being tracked in each country

Data collection for the stepped-wedge cluster randomized trial will be cross-sectional every month. Country teams will leverage existing data collection systems to gather data on primary outcomes. Specifically, teams will use ANC registers at the health facility and client cards/patient files as primary data sources. They will also collect information on cases referred to a higher level of treatment (e.g. district health hospital) for further management, where available. Secondary outcomes or indicators of interest (see Table 3) vary across each country.

**Table 3:** Qualitative methods and stakeholder groups by country

	Burkina Faso	India	Rwanda	Zambia
Pregnant women who seek ANC at selected facilities or within the selected facilities’ catchment areas	IDIs and FGDs	IDIs	FGDs	FGDs
Husbands/partners or mothers-in-law (household decision maker) from the selected catchment areas	IDIs	IDIs	FGDs	
Selected community leaders and/or champions for maternal health	IDIs			FGDs
Community health workers rendering services within catchment area of selected health facilities	IDIs and FGDs	FGDs		
Health workers providing ANC services at selected facilities or within catchment area of the selected facilities	IDIs	FGDs	FGDs* & IDIs	FGDs* & IDIs
Health facility managers of selected facilities		IDIs	IDIs	IDIs
Government health officials/program managers responsible for maternal health programs at district/state or national level	IDIs	IDIs	IDIs	

*IDIs* in-depth interviews, *FGDs* focus group discussion

*FGDs with health workers using the digital tool during the formative phase

Additional outcomes will be measured using data collected through exit interviews (Rwanda and Zambia) and nested cohort study interviews (India and Burkina Faso) with pregnant women. This component will aim to assess the quality of ANC service provision (receipt of recommended evidence-based services), acceptability, and experience of care. To recruit women, health workers may conduct the first selection of potential participants while the country team will then follow up and gather informed consent. Health workers will not receive any remuneration for taking part in the study; therefore, they do not have an incentive to recruit participants. During the consent procedure it will be clearly explained to the women that participation is voluntary, and that their care will not be affected by their decision.

### Qualitative data (acceptability of the ANC package)

To understand whether the adapted ANC packages are acceptable to and create value for women and health workers as well as their experience of care, we will collect and analyse qualitative data. Qualitative components will entail in-depth interviews (IDIs) and focus group discussions (FGDs) with purposively identified participants from stakeholder groups. Questions for the FGDs and the IDIs have been developed on the basis of the theoretical framework of acceptability to gauge women’s and health workers’ affective attitude and perceived effectiveness towards the adapted ANC package as well as their understanding (intervention coherence) of it and the sense of self-efficacy when delivering the package [21]. The stakeholder groups (see Table 4) . Additionally, qualitative data will explore the potential conditions for sustainability and scale up.

Country teams will conduct facility assessments at all the study sites using a standard process evaluation form for monitoring. The evaluation form will include items for measuring the readiness of study facilities in terms of supplies and equipment, in addition to training and supervision needs. The implementation of the updated adapted ANC package will be further documented to understand to what extent the co-interventions were performed as intended and factors that inhibited or promoted effectiveness, allowing for iteration where necessary, using the adapted version of WHO Programme Reporting Standards [30]. The analysis of these data will measure the extent of fidelity to the implementation strategy and explore its association with study outcomes.

Costing data will also be gathered during the cohort study exit interviews with pregnant women. The cost per contact will be estimated through exit interviews with beneficiaries as part of the main evaluation. Direct costs are anticipated to include all out-of-pocket costs incurred in seeking ANC, including consultation fees, transportation costs, medicine and other supply costs. Indirect costs will additionally be assessed, including wages lost and childcare costs as a result of time spent seeking ANC. Additionally, economic costs incurred by implementing partners will be tracked prospectively.

To determine the full spectrum of costs to the health system, a range of methods will be employed, including (1) tracking marginal commodity costs (through implementation research partners and WHO program records); (2) understanding infrastructure costs, including buildings and capital equipment; and, in Burkina Faso and India only, (3) assessing health worker's time.

To support the measurement of health worker time, teams will conduct observations of health worker's efforts during ANC service delivery, followed by IDIs with the health workers observed. Observations and interviews will be done at two time points in the same geography: (1) prior to guideline introduction (baseline) and (2) once the revised guidelines have been introduced and the program stabilized. Data collection will be carried out by implementation research staff using (a) structured observation sheet per type of ANC patient observed and (b) an IDI guide. The latter will be implemented as part of IDIs with health workers, who were observed and seek to understand their perceptions of time allocation on the basis of different ANC contacts and patient risk profiles. A total of 480 observations will be conducted during the baseline period and 480 observations during the intervention period across both states. All pregnant women receiving ANC services on the day of observations will be eligible to be observed. For the observations, verbal consent will be obtained from the health worker and pregnant women.

### Study population and sample size

For the stepped-wedge cluster randomized trial, data will be collected cross-sectionally from facility ANC registers (modified for this study). To ensure data quality across health facilities, an orientation for improving quality or recording and reporting will be organized across all study sites at the beginning of the study.

For the nested cohort component of the study, the pregnant women who seek ANC for the first time at the selected health facilities or at public health platforms within their catchment area will be approached and invited to participate in the study. The cohort recruitment will be closely monitored and mitigation efforts, such as additional recruitment and extension of enrolment time to ensure that the sample size is not negatively impacted, will be employed.

#### For stepped-wedge cluster randomized trial (India)

For estimating the sample size for the stepped-wedge cluster randomized design, we utilized one of the primary indicators: proportion of pregnant women who were screened for syphilis during their pregnancy. The current estimates for this indicator are approximately 20% in both Tamil Nadu and Assam. Considering 20% as the baseline, and our study having 80% power to detect a minimum change of 15%, alpha error of 5%, cluster size of 100 per period, intracluster correlation coefficient (ICC) of 0.1 and cluster autocorrelation coefficient (CAC) of 0.8, the total sample size computed is 4800 women, or 800 per period. To account for issues around data loss (incomplete entries, errors in entries, etc.), we have further inflated this computed sample size by 20%. This results in a total sample of 5760 women, or 960 per period.

#### For stepped-wedge cluster randomized trial (Burkina Faso)

To estimate the sample size for the randomized staggered-corner cluster design, the country team used one of the main indicators, that is, the proportion of pregnant women who made the first contact before 12 weeks of pregnancy. The current estimate for this indicator is approximately 40%. In total, the sample size is 1600 women per period on the basis of the following assumptions:

- 80% power
- An ICC of 0.1
- Detect a ‘true’ proportion difference of 10%.

The team will need 1600 women per period. Accounting for missing data, the team will increase the size of the sample by 20%, which brings us to a minimum of 1920 women per period, or 960 women to be surveyed per region. For each study period, we will have 1920 (60 women × 4 health facilities × 4 districts × 2 regions) or 240 women per health district. The total sample for Burkina Faso will be 11 520 women who come for ANC.

In both countries, data from 30 pregnant women who seek ANC for the first time either at the selected health facility (or in its catchment area) will be abstracted from the ANC registers during each month (see Table 5 for more details).

#### For nested longitudinal data collection (India)

For estimating the sample size for the nested longitudinal study, the research team considered a secondary outcome indicator, that is, proportion of pregnant women whose blood pressure, weight and Hb are checked at least once in each trimester. As there are no reliable estimates for this indicator from either Tamil Nadu or Assam, we assumed it is approximately 50%. For our study to detect the relative risk within 20% of true value, which we assumed to be 1.25 (*P*1 = 62.5%), the computed sample size is 124. To account for clustering by health facility, we considered a design effect of 1.5, which leads to a sample size of 186. To account for loss to follow-up and the probability of some participants shifting to the private sector during the course of their pregnancy, we further inflated the sample by 25%, which leads to a sample size of 233. The country team rounded it up to 240 to equally distribute the sample across four health facilities of each arm (60 per health facility). Therefore, we will follow a total of 480 pregnant women, 240 during three consecutive non-intervention periods and 240 during three consecutive intervention periods through their pregnancy.

#### For nested longitudinal data collection (Burkina Faso)

To estimate the sample size for the nested longitudinal study, we considered that only 81.7% of pregnant women attended at least one ANC contact [31]. The research team will consider an improvement of 10% with the intervention, that is, *P*1 = 91.4% and exposed/unexposed ratio = 1. The team considered a level of confidence of 95% and a power of 80%. The calculated sample size is 400. Considering the grouping by health facility, we considered a cluster effect of 1.5, which leads to a sample of 600. Accounting for the loss to follow-up of certain women, the country team further increased the sample by 10%, which leads to a sample size of 660 women (330 in each arm). The first 44 pregnant women who will come to each selected health facility for ANC after the start of the enrolment phase will be approached and invited to participate in the study.

### Formative phase – Rwanda & Zambia

Country teams will interview participating ANC health workers and supervisors from the selected health facilities. Teams will conduct two FGDs with participating health workers (two per facility) from the health centres. These FGD will enable teams to understand user perceptions as well as enablers and barriers to implementing the country-adapted ANC digital module and its teaching and technical support mechanisms. An additional FGD or IDI will be held with the supervisors of the same health centres to gather their perceptions on the supervisory and mentoring tools developed for the country-adapted ANC digital module’s implementation in the demonstration phase.

### Demonstration phase – Rwanda & Zambia

At 5% significance, with 14 facilities we are powered (80%) to detect an approximate 20% difference in the proportion of women who complete their first contact in the first trimester with 100 women per facility per period (4 months). Baseline data in Table 1 present that on average, approximately 30% of women attend their first contact in the first trimester, and we assume an ICC of 0.1 and a CAC of 0.6 between both groups.

To account for possible data loss, we have increased the sample size by 20%. Therefore, we will recruit 120 observations per facility per period. This would yield a sample size of 1680 per period (840 per cluster, 30 per facility per month); for a total sample of 10 080 women attending their first ANC contact over the course of 24 months of data collection.

A summary table of the planned data collection processes, sample size and data sources are available in Table 5.

### Data analysis of quantitative data

A statistical analysis plan will be developed that describes in detail how the study data will be organized and analysed. In general, health facility and participant characteristics at baseline will be described according to the appropriate summary statistics, example proportions for categorical data, and mean, standard deviation, standard error and interquartile range for continuous data. Chi-squared test will be used to compare proportions for categorical variables and *t*-test will be used to compare means for continuous variables across facilities, clusters, and districts.

Impact on adherence to the adapted ANC package by health workers will be evaluated by comparing primary parameters (varies by country) at baseline prior to implementation to when the adapted ANC package is fully implemented. Hypothesis for comparing the proportions can be expressed formally as:

$$H0: \, p1 = p2 \, vs \, H1: \, p1 \, < > \, p2$$

where p1 and p2 are respective proportions at baseline and after ANC package implementation.

Hypothesis testing at *P* = 0.05 significant level will be conducted by regression analysis using generalized linear models (GLM) to estimate both the risk difference and relative risk. The GLM model for primary analysis will allow for adjustment for correlation due to clustering across time periods.

All descriptive and statistical analyses, including survey data, will be performed using Stata SE version 15.1.

### Data analysis of qualitative data

Qualitative data from IDIs and FGDs will be audio recorded, transcribed and translated from local languages to French or English, as needed. Transcripts will be organized into thematic areas using a content analysis approach. Thematic analysis approach was identified, given its accessibility to researchers across multiple disciplines, to identify, analyse and report patterns and themes within the data [32]. The thematic analysis will be performed according to the following steps: organizing the data; generating categories, themes and patterns; testing emergent hypotheses and searching for alternative explanations. In Rwanda and Zambia, this will be done for both the formative and demonstration phases.

### Ethical considerations

The research team will take necessary measures to ensure the safety and wellbeing of the participants (health workers and pregnant women) and to ensure that they are not harmed by this study. The protocols have been approved by the WHO ethics committee and the ethics committee of each country.

## Discussion

Adverse health outcomes can be prevented by quality healthcare during pregnancy and childbirth [3, 4]. Furthermore, women themselves identify ANC as a key opportunity for receiving relevant and timely information and evidence-based clinical interventions and tests, as well as support at this critical time in the course of their lives [5, 6, 33]. To improve ANC service provision, WHO launched comprehensive guidelines on routine ANC for pregnant women and adolescent girls [9]. Ensuring uptake of WHO’s evidence-based guidelines, such as the ANC recommendations, will be key to reaching the United Nations Sustainable Development Goals (SDGs), specifically SDG3: to ‘ensure healthy lives and promote well-being for all at all ages’ [34]. Supporting the four study countries in implementing WHO’s ANC guidelines will influence their achieving SDGs targets.

Indian, Burkinabe, Rwandan and Zambian national governments have prioritized ANC and are committed to improving its quality. Since 2018, the four MoHs, supported by WHO, have led the adaptation of the 2016 ANC recommendations to country/state settings, aligning their ANC policies. These efforts represent a novel approach led by national governments, which after adapting their ANC policies on the basis of the WHO recommendations, identified the need to better understand how to implement their updated ANC service packages. This led to the development of the New antenatal care model in Africa and India (NAMAI) study, employing implementation science to design and test the packages’ implementation. The COM-B model and theoretical framework for acceptability will be employed to learn how best to design co-interventions and implement the updated national/state ANC policies. For Burkina Faso and India, a vision of success is, ultimately, understanding how to sustainably deliver and support the scale up of an updated, quality, costed, and adapted ANC package. In Rwanda and Zambia, a vision of success is, ultimately, understanding how to employ a digital intervention to sustainably deliver and support the scale up of an updated, quality, costed, and adapted ANC package and which would be integrated within the digital ecosystem [23].

Results from this study aim to build the global evidence base on how to implement quality ANC across different settings and inform pathways to scale, which ultimately will lead to stronger health systems with better maternal and perinatal outcomes. The study also seeks to contribute to the field of implementation science; to our knowledge, it is the first large-scale implementation research trial to understand the implementation of WHO ANC recommendations in LMICs. It presents an opportunity to explore the application of this framework in a different context and identify potential areas for theoretical and methodological refinement.

Moreover, we seek to strengthen links with newly released WHO recommendations on maternal and newborn care for a positive postnatal experience to improve learnings and implementation [35]. Findings from this study will further inform translation of WHO’s maternal and perinatal health (intrapartum and postnatal) recommendations into policy and practice and a global evidence base for improving service quality within integrated health systems. Study learnings will be critical to standardize approaches to adapting and implementing WHO evidence-based recommendations, tailored to country setting, including digital innovations. The governments will have more implementation evidence, tools and know-how to adopt and plan for national scale up, accelerating efforts to reach the SDGs.

## Data Availability

Not applicable.

## Appendix

See Tables 4 and 5.

**Table 4:** Secondary outcomes by country

	Burkina Faso	India	Rwanda	Zambia
Ultrasound scan at ANC-1 or before 24 weeks	X	X	X	X
HB check at first ANC contact (screening for anaemia)	X			
Tested/screened for maternal infections (TB, malaria, HIV and ASB) at first ANC contact		X		
Blood pressure and weight checked at least once during pregnancy/in each trimester	X	X		
IFA consumption for 90+ days		X		
Preventive anthelmintic treatment		X		
Gestational weight gain during pregnancy		X		
Tested for gestational diabetes mellitus (GDM) in pregnancy		X		
More than 8 ANC contacts			X	
4 ANC contacts			X	
Intermittent preventive treatment in pregnancy (IPTp) for malaria (at least three doses)	X			X
Women diagnosed with maternal infections (HIV, hepatitis) initiated on treatment	X			
Women diagnosed with GDM in pregnancy initiated on treatment	X			

X denotes which indicator is being tracked in each country

**Table 5:** Data collection summary – quantitative

Data collection	Data source	Sample size	Sampling
Cross-sectional ANC service data	Summary aggregate data from facility registers	Once per month per study health facility	Collected on monthly basis from all study health facilities
	ANC details of pregnant women as recorded in study health facility registers	Burkina Faso and India: • Total 11 520 pregnant women • Total 5760 pregnant women per region/state • 1920 per study period • 120 per health facility per period • 30 per health facility per month	All pregnant women who seek ANC for the first time at the study health facilities
		Rwanda and Zambia: • Total 10 080 pregnant women • 1680 per study period • 120 per health facility per period • 30 per health facility per month	
	Referral details of pregnant woman (if referred during first ANC contact)	All pregnant women who were referred during first ANC contact	Pregnant women who were referred during first ANC contact
Nested longitudinal cohort data (Burkina Faso & India only)	Cohort of pregnant women	Burkina Faso: • Total 660 pregnant women • 330 pregnant women per arm (non-intervention versus intervention periods)	Pregnant women who seek ANC for the first time during the first trimester at the study health facilities or at public health platforms within catchment areas of selected health facilities
		India: • Total 960 pregnant women • Total 480 pregnant women per state • 240 pregnant women per arm (non-intervention versus intervention periods)	
Cross-sectional survey data (Rwanda & Zambia only)	Exit interviews with pregnant women	• Total 560 interviews with pregnant women • 280 interviews post ANC package co-intervention implementation • 280 interviews post ANC digital tool co-intervention implementation • 10 interviews per health facility per month during 4 study periods	Pregnant women who seek ANC for the second time during the first trimester at the study health facilities

## Abbreviations

WHO AFRO: WHO Africa Regional Office
ANC: Antenatal care
MoH: Ministry of Health
SDGs: Sustainable Development Goals
QES: Qualitative evidence syntheses
COM-B: Capability Opportunity Motivation-Behaviour
BCW: Behaviour change wheel
EPOC: Cochrane effective practice and organization of care
ToT: Training of trainers
IFA: Iron and folic acid
Hb: Haemoglobin
FGD: Focus group discussion
IDI: In-depth interview
GDM: Gestational diabetes mellitus
HIV: Human immunodeficiency virus
TB: Tuberculosis
IPTp: Intermittent preventive treatment in pregnancy

## Footnotes

1 In comparison with the national average.

2 The implementation research partners are as follows: Institut de Recherche en Sciences de la Santé (IRSS) - Burkina Faso, Jhpiego – India office, University of Rwanda School of Public Health, Population Council – Zambia office.

3 The MoH-selected partners are: 1000 Hills Solutions in Rwanda and BlueCode Systems in Zambia. The partner that developed the generic module is Ona Systems Inc. – Kenya.
